# Peripheral Artery Disease as a Risk Factor for Myocardial Infarction

**DOI:** 10.7759/cureus.15655

**Published:** 2021-06-15

**Authors:** Erick Leonel Pérez Mejias, Sila Mateo Faxas, Nicole Tejeda Taveras, Abdul Subhan Talpur, Jitesh Kumar, Maria Khalid, Suraj K Aruwani, Dua Khalid, Haya Khalid, Sidra Memon

**Affiliations:** 1 Internal Medicine, INTEC Education College, Shah Alam, MYS; 2 Internal Medicine, Universidad Iberoamericana, Santo Domingo, DOM; 3 Medicine, Liaquat University of Medical and Health Sciences, Jamshoro, PAK; 4 Internal Medicine, Ghulam Muhammad Mahar Medical College, Sukkur, PAK; 5 Internal Medicine, Shaheed Zulfiqar Ali Bhutto Medical University, Islamabad, PAK; 6 Internal Medicine, Jinnah Sindh Medical University, Karachi, PAK

**Keywords:** peripheral artery disease, risk factor, myocardial infarction, association, fatal myocardial infarction

## Abstract

Introduction: Atherosclerosis contributes to the underlying pathophysiology for peripheral arterial disease (PAD), coronary artery disease (CAD), and cerebrovascular disease. Several studies have been conducted to demonstrate PAD as a major risk factor for cardiovascular (CV) events, however, the regional data are limited. This study aims to highlight PAD as a major risk factor in CV events in a local setting.

Methods: In this longitudinal study, 400 hypertensive patients with a confirmed diagnosis of PAD were enrolled from the outpatient department of the cardiology unit. Diagnosis of PAD was made using the ankle brachial index (ABI). ABI less than 0.9 was labeled as participants with PAD. Another group of 400 without PAD was also enrolled as the control group from the outpatient department of cardiology unit. Patients were followed up for 12 months or for the development of myocardial infarction (MI).

Result: Participants with PAD had a significant increased risk of total MI events with a relative risk (RR) of 1.67 (confidence interval, CI 95%: 1.05-2.66; p-value: 0.02). The RR for fatal MI was 2.62 (CI 95%: 0.94-7.29; p-value: 0.06) compared to the participants without PAD, however, it was not significant.

Conclusion: This study has focused on the risk factors of PAD and has suggested that the patients who have any of the mentioned risk factors should be treated with caution under strict instructions given by doctors. A variety of treatment options is available, but the initial changes should be made in the lifestyle of these patients, making sure the risk factors are being treated.

## Introduction

Atherosclerosis contributes to the underlying pathophysiology for peripheral arterial disease (PAD), coronary artery disease (CAD), and cerebrovascular disease [1]. This condition is characterized by a diseased endothelium, low-grade inflammation, lipid accumulation, and plaque formation within the intima of the vessel wall [2]. Rupture of the plaque can provoke superimposed atherothrombosis as well as occlusion of the vessel wall, which leads to the development of cardiovascular (CV) events including myocardial infarction (MI), stroke, limb ischemia, and CV death [3]. Disease in more than one arterial bed is associated with the worst prognosis [4]. However, the prognosis can be improved through secondary preventive measures, with lifestyle changes and medicinal control of modifiable CV risk factors [5].

Several studies have been conducted to demonstrate PAD as a major risk factor for CV events. According to the 2016 study, the global burden of CV disease due to PAD was estimated to be 25.6% and 1.7% of the overall global burden of the disease [6]. Reduced quality of life (QOL) was predominantly associated with patients with PAD because of the functional limitations caused by the symptoms. In the European Union and the United States National Health and Wellness Survey, it was reported that patients with PAD had a lower mental and physical health-related QOL [7]. Major adverse CV events are associated with PAD. Blin et al. revealed that within a year of MI, PAD was the most important factor in predicting the risk of re-infarction, stroke, or transient ischemic attack [8].

This study aims to highlight PAD as a major risk factor in CV events. This further emphasizes the coping strategies and follows the secondary measures to improve the QOL in patients with PAD.

## Materials and methods

This longitudinal study was conducted from April 2019 to February 2021 in the cardiology unit of a tertiary care hospital. We enrolled 400 hypertensive patients with a confirmed diagnosis of PAD from the outpatient department of cardiology via consecutive convenient non-probability sampling. Diagnosis of PAD was made using the ankle brachial index (ABI). ABI less than 0.9 was labeled as participants with PAD. Another set of 400 participants without PAD were also enrolled from the outpatient department as the control group.

The patient’s characteristics such as age, gender, history of smoking, blood pressure, previous and family history for MI were noted in a self-structured questionnaire. Patients were followed up for 12 months or until the development of MI, whichever came first. MI was diagnosed based on symptoms, electrocardiogram (ECG), and cardiac enzymes. 

Participants lost to follow-up in groups with and without PAD were 47 and 43, respectively. Only participants who completed the study were included in the final analysis. Statistical analysis was done using the Statistical Packages for Social Sciences (SPSS) version. 23.0 (IBM Corporation, Armonk, New York, USA). Continuous variables were analyzed via descriptive statistics and were presented as mean and standard deviation (SD) while categorical variables were presented as percentages and frequencies. Relative risk (RR) was calculated via an online calculator (MedCalc Software Ltd., Acacialaan 22, 8400 Ostend, Belgium) using a 95% confidence interval (CI). A p-value of less than 0.05 meant that there is a difference between the two groups and the null hypothesis is void.

## Results

Seven hundred and ten (710) participants completed the study, 353 and 357 participants with and without the PAD group, respectively. There was no difference in demographics and the risk factor profile between the two groups (Table 1).

**Table 1:** Characteristics of the study participants. BMI, body mass index; MI, myocardial infarction; NS, non-significant; PAD, peripheral artery disease; SD, standard deviation

Characteristics	Participants with PAD (n= 353)	Participants without PAD (n= 357)	p-value
Age in year (mean ±SD)	47 ± 11	48 ± 10	0.20
Male (%)	201 (56.9%)	198 (55.5%)	0.69
Smoking (%)	121 (34.2%)	127 (35.5%)	0.71
Diabetes (%)	178 (50.4%)	182 (50.9%)	0.88
Hypercholesterolemia	213 (60.3%)	221 (61.9%)	0.66
BMI greater than 25 kg/m^2 ^(%)	98 (27.7%)	101 (28.2%)	0.87
Previous history of acute MI (%)	25 (7.0%)	17 (4.7%)	0.19
Family history of acute MI (%)	10 (2.8%)	12 (3.3%)	0.68

Participants with PAD had a significant increased risk of total MI events with a RR of 1.67 (CI 95%: 1.05-2.66; p-value: 0.02). The RR for fatal MI was 2.62 (CI 95%: 0.94-7.29; p-value: 0.06) compared to the participants without PAD, however, it was not significant (Table 2).

**Table 2:** Classification of MI in participants with and without PAD. NNH, number needed to harm; MI, myocardial infarction; PAD, peripheral artery disease

MI	Participants with PAD (n=353)	Participants without PAD (n=357)	Relative risk (CI 95%)	NNH	p-value
Total MI	43 (12.1%)	26 (7.2%)	1.67 (1.05-2.66)	20.4	0.02
Non-fatal MI	30 (8.4%)	22 (6.1%)	1.37 (0.81-2.34)	42.8	0.23
Fatal MI	13 (3.6%)	4 (1.1%)	2.62 (0.94-7.29)	43.8	0.06

## Discussion

In our study, both groups did not show any significant differences in their demographics. Risk factors namely smoking, diabetes, hypercholesterolemia, BMI greater than 25 kg/m2, previous and family history of acute MI did not vary significantly.

Our results demonstrated that patients with PAD were reported to be at a greater risk of total and fatal MI, compared to those who did not have PAD. However, the trends were not significant for fatal MI. This may be due to a limited number of fatal events. These results are supported by the fact that PAD is known to be a potential cause of cerebrovascular and CV episodes along with higher mortality rates [9-11]. PAD is known to be associated with acute MI [11-13]. Our results have been supported by other studies [14-18] that stated that 9% of acute MI had a prior history of PAD, and PAD was mostly reported among those at a higher risk of having underlying diseases like hypertension, diabetes, and stroke [14, 19-21].

The major risk factors for PAD are smoking, hypertension, hyperlipidemia, diabetes, obesity, and a family history of vascular disease. The National Health and Nutrition Examination Survey of 1999-2000 examined 2000 patients with PAD and concluded that approximately 95% of them had at least one of the mentioned risk factors, and up to 70% had more than two risk factors [22]. PAD patients who had diabetes and smoking habits were 2.5 times at an increased risk of morbidity and mortality [23]. Diabetes is known to be linked with accelerated atherosclerosis and increased episodes of cardiac events [24]. Novel risk factors include increased inflammatory markers such as C-reactive protein, fibrinogen, and plasma homocysteine [25]. Hyperhomocysteinemia is also an independent risk factor for PAD [26]. There are various strategies related to the treatment of PAD, including lifestyle modification, medical management, endovascular therapies, and surgical interventions. These strategies help in the management of claudication symptoms and secondary prevention of CV complications [27].

To the best of our knowledge, this is the first study from this South Asian region to study PAD as a risk factor for MI. However, since the study was conducted in a single center, care should be taken while inferring the result to a larger population. This study has focused on the risk factors of PAD and has suggested that the physician should treat the patients who have any of the mentioned risk factors with caution under strict instructions.

## Conclusions

Peripheral arterial disease is known to potentially cause a global burden by leading to MI. However, our study is of the idea that by tracing the risk factors, PAD could be treated even before the onset of complications. Therefore, proper screening and safe treatment options could help in the long term. Moreover, to ensure the effectiveness of the treatment, check-ups should be done at regular intervals.
